# Prion Protein Expression and Processing in Human Mononuclear Cells: The Impact of the Codon 129 Prion Gene Polymorphism

**DOI:** 10.1371/journal.pone.0005796

**Published:** 2009-06-04

**Authors:** Christiane Segarra, Sylvain Lehmann, Joliette Coste

**Affiliations:** 1 Etablissement Français du Sang de Pyrénées Méditerranée, Montpellier, France; 2 Institut de Génétique Humaine, UPR1142 CNRS, /CHU Montpellier/UM1 Montpellier, Montpellier, France; Columbia University, United States of America

## Abstract

**Background:**

So far, all clinical cases of new variant Creutzfeldt-Jakob disease (vCJD), thought to result from the Bovine Spongiform Encephalopathy (BSE) prion agent, have shown Methionine–Methionine (M/M) homozygosity at the M129V polymorphism of the PRNP gene. Although established, this relationship is still not understood. In both vCJD and experimental BSE models prion agents do reach the bloodstream, raising concerns regarding disease transmission through blood transfusion.

**Methodology/Principal Findings:**

We investigated the impact of the M129V polymorphism on the expression and processing of the prion protein in human peripheral blood mononuclear cells (PBMCs) from three blood donor populations with Methionine-Methionine (M/M), Valine-Valine (V/V) and M/V genotypes. Using real-time PCR, ELISA and immunoblot assays we were unable to find differences in prion protein expression and processing relating to the M129V polymorphism.

**Conclusions/Significance:**

These results suggest that in PBMCs, the M129V PrP polymorphism has no significant impact on PrP expression, processing and the apparent glycoform distribution. Prion propagation should be investigated further in other cell types or tissues.

## Introduction

Transmissible spongiform encephalophathies (TSEs or prion diseases) are fatal neurodegenerative disorders, of which the main human form is Creutzfeldt–Jakob disease (CJD) [1], [2]. Importantly, a new variant of CJD (vCJD) has recently emerged [3], believed to result from oral exposure to Bovine Spongiform Encephalopathy (BSE). Although the exact nature of the infective agent remains controversial, many believe that infectivity is closely associated with the accumulation of a pathological misfolded protein, PrP^Sc^ (Scrapie Prion Protein), derived from the normal cellular protein PrP^C^ [4]. PrP^C^ and PrP^Sc^ isoforms share the same primary structure [5]: a single disulfide bond [6], two complex-type N–Linked oligosaccharide chains [7], [8], and a C-terminal glycosyl phosphatidylinositol (GPI) anchor [9]. The difference between the isoforms relates to their secondary structure: PrP^C^ is rich in α-helices, while PrP^Sc^ has a higher content of β-pleated sheets [10]–[12]. During prion propagation, the increase in the level of infectivity is associated in most cases with the accumulation of the misfolded PrP^Sc^ protein, believed to convert PrP^C^ through a pathological conformational rearrangement [13]. Although the mechanism explaining this transconformation remains unclear, two different models have been proposed: nucleation-polymerization and template-assisted conversion [14], [15]. Both involve the recruitment of normal prion protein PrP^C^. Biochemical studies in cultured cells demonstrate that conformational rearrangement of PrP^C^ can occur in the absence of glycosylation [16] and that N-glycan chains can modulate the *in vitro* conversion of PrP^C^ into PrP^Sc^-like molecules [17]. In the normal brain, PrP^C^ processing includes proteolysis of the full-length protein to generate a main N-terminal truncated protein named the C1 fragment. However, proteolysis can also generate an additional, longer C-terminus protein –the C2 fragment - that accumulates in prion-infected brain in particular. [18].The C2 fragment only accumulates as a resistant truncated protein in CJD-affected brains. This raises questions about whether this truncated form influences the possibility of developing the illness. Moreover, studies have described the accumulation of under-glycosylated, full-length and N-Terminal truncated PrP as a fingerprint for prion disease [19].

The PRNP gene, on the short arm of human chromosome 20, encodes the prion protein [20]. Some polymorphisms of the PRNP open reading frame (ORF) seem to be involved in susceptibility to different forms of CJD (iCJD, gCJD, sCJD, vCJD) [21]. This is illustrated by the fact that 100% of the clinical vCJD cases thus far have been Methionine –Methionine homozygous at the M129V polymorphism (129-M/M) [22], yet only 40% of the Caucasian population carry this genotype. Importantly, in classical CJD patients, PrP^Sc^ accumulates in neuronal, [23], [24] follicular dendritic cells, and muscle [25], but for vCJD patients, PrP^Sc^ also accumulates in lymphoid tissue, including the tonsils and appendix [26]–[29]. This distribution raised concerns about blood transfusion from the very beginning of the epidemic. Since then, studies by Houston F. *et al* [30] have demonstrated that prion transmission by blood transfusion occurs experimentally in sheep infected with BSE. In humans, three UK vCJD patients had previously been transfused with red cells donated by individuals who subsequently developed vCJD 1.5–3.5 years after donation. All three transfusion-recipient patients carried the 129-M/M genotype. A fourth recipient of red cells from a donor who later developed vCJD, died from unrelated causes. Post mortem investigation in this case found abnormal prion accumulation in the spleen and in one cervical lymph node, but not in the brain [31]–[33]. Recently, a person with hemophilia who died of a condition unrelated to vCJD, only showed evidence of infection with the abnormal prion protein in the spleen at post mortem. In these cases without clinical signs, both patients were heterozygous 129-M/V. This bears in mind the sub-clinical CJD cases with 129-V/V recently described in a retrospective study of appendix samples [34]. Polymorphism at position 129 could, therefore, not only influence susceptibility to prions, but also impact the incubation time or clinical onset of the disease. One possible mechanism to explain this relation would be the consequence of 129 polymorphism on PrP^C^ expression and processing that are critical for prion propagation [4], [16], [17], [35]. Given the concerns regarding vCJD transmission through blood transfusion, studying PrP^C^ in blood seems particularly relevant. Indeed, PrP^C^ distribution determined by ELISA and flow cytometry, shows that plasma mononuclear cells and platelets express PrP^C^, although contradictory results were obtained for red cells and granulocytes [36], [37].

In order to investigate a possible relationship between the M129V polymorphism and susceptibility to vCJD and PrP^C^ expression, we compared three blood donor populations of different genotypes: M/M, M/V, and V/V at position 129. We analyzed their expression of total PrP^C^ (mRNA, protein) and PrP^C^ isoforms (glycosylated and truncated proteins) in peripheral blood mononuclear cells (PBMCs).

Our results showed that in these cells, PrP expression and processing did not determine susceptibility to prion disease.

## Materials and Methods

### Blood Samples

After having obtained informed consent in writing from each blood donor, in compliance with the French law (code de la santé publique article L.1243-3), whole blood was taken in EDTA collection tubes. Given the well known distribution of codon M129V genotypes (41% M/M, 49% M/V and 10% V/V) in the French population, the study did not require the approval of the Bioethical Review Board. We genotyped a total of 119 donors in order to obtain a statistically representative V/V population.

### Isolation of peripheral blood mononuclear cells (PBMCs)

In order to avoid bias due to physiological variation in the percentage of granulocytes (40 to 70%) in human blood, we examined only purified mononuclear cells. Indeed, preliminary studies performed on leukocyte populations (data not shown) indicate a lower PrP^C^ mRNA expression in granulocytes than mononuclear cells. Consequently, we only purified mononuclear cells from blood by centrifugation through a Ficoll-Hypaque gradient (Pharmacia, 91 898 Orsay, France). In brief, whole blood diluted Vol./Vol. with phosphate-buffer saline (PBS) was carefully layered upon Ficoll and spun at 700 g. Mononuclear cells were harvested from the interface of the plasma-platelets layer and the Ficoll, washed in PBS and suspended in the same buffer. The cell suspension was analyzed in triplicate, on the ABX (Beckman Coulter, 95942 Roissy, France) hemocytometer for total cell number and mononuclear cell purity level. After elimination of PBS, aliquots of 10^6^ PBS-cleared PBMCs were stored at −80°C.

### RNA extraction

Total RNA was isolated (High Pure RNA isolation kit, Roche Diagnostics, 38240 Meylan France) from 10^6^ PBMCs, according to the manufacturer's instructions. Briefly, cells were lysed by solubilizing them in lysis/binding buffer optimized for RNA extraction (4,5M guanidin hydrochloride, 50 mM Tris-HCl and 30% Triton X-100, pH 6.6). Homogenized lysate was then transferred to a filter tube for binding to the fiberglass filter. After centrifugation at 10 000 g for 15 sec, residual contaminated DNA was digested by DNase I (180 U/tube) applied directly to the fiberglass filter and incubated for 15 min. After washing three times - in order to eliminate enzymes and cellular impurities - purified RNA was eluted with 50 µl elution buffer (nuclease-free sterile water), and immediately stored at −80°C.

As the PRNP ORF sequence is located on only one exon, to exclude potential contamination by genomic sequences, we treated total RNA extracts with RNase-free DNase before amplification. Samples were then controlled for the absence of genomic sequence by performing PCR directly on the RNA extracts, thus avoiding the reverse transcription step.

### cDNA synthesis

The first cDNA strand was synthesized by reverse transcription (R.T. at 42°C for 1 hour) from 5 µl of RNA (preheated for 7 min at 70°C) in the presence of hexanucleotides (7 µM), dNTPs (800 µM each), 60 U Rnasin Ribonuclease Inhibitor (Promega, 69260 Charbonnières France) and 300 U M-MLV-RT (Promega) in a final volume of 35 µl. cDNA was then purified with “High Pure PCR Product Purification Kit” (Roche Diagnostics) according to the manufacturer's instructions. The test principle was the same as previously described for RNA extraction; the only difference concerned the lysis buffer, which was adapted for DNA products. Finally, cDNA was eluted with 50 µl elution buffer, supplemented with salmon sperm DNA (10 ng/µl final), and kept at −20°C.

### Real-time PCR

#### Standard curve preparation

First, RNA was produced by *in vitro* transcription with AmpliScribe T7, T3 and SP6 High Yield Transcription Kit (Epicentre/Tebu 78610 Le Perray-en-Yvelines France) according to the manufacturer's instructions. The ORF sequence (759 pb) of the human prion gene - inserted into a pcDNA3 plasmid - was used as the reaction template. Second, four one-in-ten serial dilutions were prepared from a stock RNA solution at 10^−11^ grams/µl (g/µl). This yielded a standard curve with concentrations ranging from 10^−12^ to 10^−15^ g/µl, corresponding to 2.33×10^6^ and 2.33×10^3^copies/reaction.

#### Primer Sequences

Primer Sequences selected for real-time PCR were those described by Dodelet *et al.* [38]. They ensure amplification of a fragment of 432 pb length, encompassing the M129V polymorphism within the human prion ORF.

#### Quantification

Quantification was carried out on a LightCycler by real-time PCR and SYBR Green I dye (Roche Diagnostics, Penzberg, Germany). Amplification assays were performed with 1 µl of cDNA in 20 µl final reaction mixture containing 2 µl LightCycler-FastStart DNA Master SYBR Green (Roche Diagnostics) and 0.6 µM of each primer (forward, nt 79 to 99 and reverse, nt 498 to 510). PCR conditions consisted of an initial denaturation at 95°C for 5 min, and 45 cycles at 95°C for 15 sec, 65°C for 5 sec, 72°C for 15 sec, 89°C for 5 sec. All samples were analyzed in duplicate. The amount of DNA was estimated after each PCR cycle by reading fluorescent dye incorporation (SYBR Green) at 530 nm in the PCR product. At the end of each run, a DNA melting step was performed and a fusion curve was recorded to control the homogeneity and specificity of the amplified DNA.

#### Analysis of the results

Readings were performed for each cycle after a fourth segment at 89°C, in order to exclude non-specific signal interference with the concentration calculation. Analysis was automatically performed by LightCycler software 4.0 as follows: after PCR completion, LightCycler software calculated the copy number of target molecules by plotting the logarithm of fluorescence versus cycle numbers and setting a baseline x-axis. From each sample, the baseline identified the cycle (crossing point, Ct) at which the log-linear signal could be distinguished from the background. Each run was analyzed by setting the noise band just above the background fluorescence. A value corresponding to F = 0.5 was systematically chosen for comparison between the runs. Regression of the x-axis crossing point of each standard with known concentrations defined the standard curve from which unknown samples were estimated. Specificity was achieved by plotting a melting curve graph based on the final PCR.

### 129 codon Genotyping

According to Teupser *et al* [*39*] the following probe pair spans the nucleic acid sequence responsible for the polymorphism: a detection probe complementary to the 129M allele and 3′-labeled with fluorescein, and the adjoining, anchoring probe 5′-labeled with Red 640. Genotyping assays were carried out in a reaction mixture containing 10 µl of amplicons and probes, at 2.5 picomoles each per reaction. Assay conditions consisted of initial denaturation at 95°C for 2 min, followed by hybridization at 42°C for 2 min and final denaturation by increasing the series to 75°C at a rate of 0.1°C with continuous reading at 640 nm. Analysis using LightCycler was performed by creating a melting profile that defined the melting temperature (Tm) of the hybrid, composed of the target DNA and the fluorescent probe pair. Tm depends on the degree of homology between the two strands, which distinguishes M129V polymorphism. At the end of the reading, LightCycler software 4 draws a melting curve, by plotting fluorescence (F) versus temperature (T), which is automatically converted to melting peaks (-dF/dT).

### Preparation of protein lysate and PNGase F-deglycosylated protein

All samples were prepared from 10^6^ PBMCs according to 2 protocols:

#### Protein lysate preparation only

Cells were dissociated and incubated on ice for 30 min in a lysis buffer containing 150 mM NaCl, 0.5% triton X-100, 0.5% sodium deoxycholate and 50 mM Tris-HCl pH 7.5, and supplemented with a protease inhibitor cocktail (Roche, 82372 Penzberg Germany). After centrifugation at 450 g for 2 min, the supernatant was harvested, added to 4× loading buffer (Invitrogen, Paisley, UK), heated at 95°C for 5 min and stored at −80°C.

#### Protein lysate preparation with PNGase treatment

Protein lysate preparation with PNGase treatment was carried out in order to reduce the heterogeneity of the PrP^C^ bands resulting from protein-bound Asn-linked oligosaccharides.

Incubation for 30 min on ice was followed by protein reduction, performed by adding 1% 2-mercaptoethanol and incubating at 90°C for 5 min. After snap cooling on ice, 2 units of PNGase F (Roche Diagnostics) were added and digestion was carried out overnight at 37°C. Then, we precipitated the proteins with 3 volumes of cooled methanol for 2 hours at −20°C. Following centrifugation at 20,000 g for 10 min, the dry pellet was suspended in lysis buffer prior to denaturation with 4× loading buffer (Invitrogen, 95613 Cergy-Pontoise France) and heated at 95°C for 5 min. Finally, samples were stored at −80°C.

#### Total protein quantification

For all samples, we quantified the lysates for total protein by Bicinchoninic Acid Protein Assay (BCA assay, Sigma, 38297 Saint Quentin Fallavier France), before freezing, according to the manufacturer's instructions.

### Determination of PrP^C^ levels

“The Enzyme Immuno Assay (EIA) Kit for the determination of PrP^C^ Protein” (SpiBio 91741 Massy France) was applied to 10^6^ PBMCs, according to the manufacturer's instructions. Briefly, we lysed cells by solubilizing them in 65 µl of extraction buffer (10 mM Tris-HCl pH 4, 100 mM NaCl, 10 mM EDTA, 0.5% IGEPAL, and 1% Deoxycholic Acid). After spinning at 10,000 g for 5 min., the supernatant was denatured by adding 4 M Urea (final concentration, diluted in Tris-HCl 10 mM pH 7.4)) and heating at 100°C for 10 min. Then the EIA, based on a double-antibody sandwich principle, was performed. The micro-well plate was coated with a monoclonal antibody specific to the prion protein (aa 144–153). The detection antibody, conjugated with acetylcholinesterase (AchE), recognized the octo-repeat region located in the N-terminus part of the PrP^C^. Readings were performed at 414 nm.

### SDS-PAGE and Immunoblotting

#### Antibodies

Four different mouse antibodies were used to span large regions of PrP: SAF32 (SpiBio, 91741 Massy, France) - which recognizes an octa-repeat region located in the N-terminus part of human PrP (human numbering 79–91), 8G8 (SpiBio) - directed against an epitope of human PrP residues 95–110, 3F4 (Signet/Proteogenix, 67412 Illkirch France) - specific for an epitope of human PrP residues 109–112, and an antibody provided from ascites fluid PRI 917 (J.Grassi, CEA Saclay, France) - spanning the COOH- terminus region of human PrP^C^ (aa: 216–221). Normalization of the expected signals was achieved using the signal obtained with monoclonal anti -α- tubulin (Sigma).

#### SDS-PAGE and Immunoblotting

Protein samples (0.5 10^6^ cells/lane i.e. 50 µg of total protein) were separated by 12% NUPAGE gel electrophoresis (Invitrogen) at 150 volts for 90 min. Proteins were then transferred to nitrocellulose membranes Hybond (Amersham biosciences, 91400 Orsay, France) for 45 min at 400 mA using the TE 22 transfer unit (Amersham biosciences). After blocking with 5% non-fat milk in PBS-0.05%Tween 20 buffer, blots were probed with the panel of selected antibodies in the dilution conditions recommended by the manufacturer. Immunoreactivity detection was carried out by a secondary anti-mouse IgG peroxydase-linked antibody, involved in an ECL reaction (ECL reagent, Amersham biosciences) and visualized on an ECL film (Amersham biosciences). Results were quantified by density readings using Sigmagel Analyzer Software (Sigma).

We prepared five protein samples for each genotype, both with and without PNGase F treatment. Samples were then analyzed using 12% SDS-PAGE separation. We normalized the samples, by migrating lysate obtained from 0.5×10^6^ cells - a total protein concentration of 50 µg +/− 10%/lane - and by comparing the 50 kDa signals obtained by anti -α- tubulin antibody blotting (not shown).

### Statistical analysis

Statistical analysis was performed by the test of mean comparison which obeys the Student-Fisher law (from Fisher and Yates, Statistical tables for biological, agricultural and medical research, Olivier and Boyd, Edinburgh). This means that the mean difference is *not* significant when the calculated absolute t value is *less* than 0.05. If the value is greater than 0.05, this represents a difference between populations.

## Results and Discussion

### Characterization of the peripheral blood mononuclear cells

One possible mechanism explaining the relationship between the M129V polymorphism and disease development, would be that the polymorphism influences PrP^C^ expression and processing critical for prion propagation [4], [16], [17], [35]. We decided to test this hypothesis on peripheral blood mononuclear cells (PBMCs) for the following reasons: the controlled and ethical ease of access to PBMCs from blood donors allowed us to collect enough samples to perform a robust analysis. Collecting several hundred human samples is virtually impossible with other tissues. Secondly, as mentioned before, blood is an important issue in the context of vCJD and our results could be important to evaluate the relative risk of PrP^Sc^ transmission in different blood components. As PBMCs express the highest level of PrP compared to platelets and red blood cells the choice seemed evident even if no experimental data have showed yet that these cells could indeed propagate prions. In any case, it is not clear how the prion agent reaches the bloodstream. Theories propose local replication in specific cell types, like dendritic cells, or secretion from extra- vascular tissues [40], [41]. Finally, we hypothesized that if the M129V polymorphism exerted a more general impact on PrP expression, it could still be observed in the PBMCs.

Quality controls of the isolated cell population indicated systematic granulocyte contamination (10% to 15%), with an acceptable variation of 5% between samples. To determine the 129 polymorphism, we used melting curve analysis to show a specific melting peak for each genotype: 129-M/M homozygous genotype peaked at 60°C, V/V homozygous at 52°C, whereas M/V heterozygous gave 2 peaks at 52°C and 60°C. The genotype distribution of the donor population of the Languedoc region, France was 42% M/M, 42% M/V and 16% V/V, values that agree with the German and the Spanish populations (Table 1) [39], [42]. Of note, a previous study on the French population observed values of 44% M/M, 46,5% M/V and 9,5% V/V) [43]. However, this study used RFLP-PCR (Restriction Fragment Length Polymorphism-Polymerase Chain Reaction), a procedure dependent on digestion efficiency.

**Table 1:** M129V polymorphism distribution.

	Met/Met^a^	Met/Val^a^	Val/Val^a^
France^b^	42%	42%	16%
Spanish (52)	40%	46%	14%
German (50)	43%	43%	14%

a values calculated from 50, 50, and 19 samples respectively per genotype.

b Languedoc region.

### PrP^C^ expression studies

Since PrP^Sc^ generation depends on PrP^C^ expression [44], we examined PrP^C^ expression in each of the three genotype populations. We first analyzed PrP^C^ mRNA levels using real-time PCR amplification and SYBR Green detection for optimal sensitivity. Samples were quantified using a standard curve composed of 4 concentrations ranging from 2.33×10^6^ to 2.33×10^3^ copies/reaction. This agreed with the linear correlation between concentration and cycle number (correlation coefficient: 1 and slope at 3.47, indicating an acceptable PCR yield at 97%). Results from the 50 M/M, 49 M/V and 19 V/V blood samples showed no significant difference between the mRNA levels of the three populations (Table 2, observed t-value less than the 0.05 critical t-value). Nevertheless, the coefficient of variation (CV) indicated a maximum of 4 fold deviation within each group. In view of the numerous steps involved in this method and the normal variation (0.5–0.7 log_10_) in technologies based on the same number of steps [45], a deviation of around 0.6 log_10_ within each group, denoted low physiological variation. The absence of significant differences in mRNA expression between the three genotypes could indicate that transcription regulation does not relate specifically to genotype. On the other hand, gene expression profiling studies of scrapie-infected brain tissue have identified numerous genes that do modify mRNA expression. Likewise, expression of proteins encoded and involved in several pathways (e.g. inflammatory reaction, proteolysis, protease inhibition, cell grown, stress, immune responses…) [46]–[49] was modified. For these reasons, we needed to confirm our results at the protein level. We determined PrP^C^ protein levels by using a commercial EIA kit, whose N- and C-terminal antibodies detect the full-length protein. As standard curves were unavailable, we compared absorbency values expressed as Absorbency Unit (AU) for each group. Results obtained from 10 blood samples from each group revealed no statistical difference (observed *t-*value less than the 0.05 critical *t*-values) in total PrP^C^ levels between the groups (Table 2). Patients with CJD - and other neurodegenerative diseases - show increased PrP^C^ expression in plasma compared to healthy control groups [50]. Our findings suggest that such over-expression in plasma does not result from PBMCs. Nor does it seem to be linked with M129V polymorphism. Moreover, our total PrP^C^ analysis conflicted with those obtained in sheep, where PBMC surface PrP^C^ expression did depend upon the genotype. Researchers found the highest levels of PrP^C^ in scrapie-susceptible VRQ/VRQ sheep, and the lowest levels in scrapie-resistant ARR/ARR genotypes [51].

**Table 2:** PrP^C^ expression and processing depending on the M129V polymorphism.

	PrP^C^ mRNA c	PrP^C^ d	PrP^C^ glycosylation^a^	PrP^C^ cleavage b	PrP^C^ cleavage b
	(copies/2000 cells)	(Absorbance Unit)	diglyc./unglyc.^e^	F.L./C2^f^	C1/C2^g^
**Met/Met**	**3.63E+05**	**1.81**	**8.86** *(SAF 32);* **3.56** *(PRI 917);* **1.72** *(8G8)*	**6.39** *(8G8);* **3.53** *(3F4)*	**2.99** *(PRI 917)*
**Met/Val**	**4.06E+05**	**2.02**	**7.25** *(SAF 32);* **3.65** *(PRI 917);* **1.83** *(8G8)*	**7.50** *(8G8);* **2.71** *(3F4)*	**2.20** *(PRI 917)*
**Val/Val**	**4.98E+05**	**2.15**	**8.87** *(SAF 32);* **3.56** *(PRI 917);* **2.09** *(8G8)*	**6.91** *(8G8);* **2.22** *(3F4)*	**2.18** *(PRI 917)*

a 13%<VC<47%.

b 3%<VC<60%.

c 52%<VC<63%.

d 15%<VC<20%.

e ratio diglycosylated PrP^C^/unglycosylated PrP^C^ calculated by image analysis of the western blot assay performed with SAF 32, PRI 917 and 8G8 antibodies.

f ratio full length unglycosylated PrP^C^/C2 fragment calculated by image analysis of the western blot assay performed with 8G8 and 3F4 antibodies.

g ratio C1 fragment/C2 fragment calculated by image analysis of the western blot assay performed with PRI 917 antibody.

### PrP^C^ glycosylation studies

**Figure 1:**
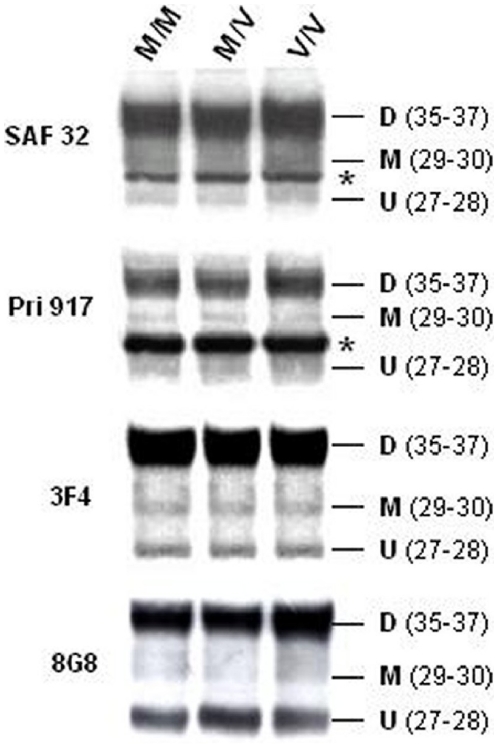
Analysis of PrPC glycosylation. Immunoblots of PrP^C^ glycoforms from the three PRNP codon 129 genotypes in PBMCs. Denatured lysates from 10^6^ PBMCs were tested by Western Blot analysis using 4 antibodies to span the large PrP region: SAF32 (79–91), 8G8 (95–110), 3F4 (109–112) and PRI 917 (216–221). The 35–37 kDa band corresponds to the M.W. of the diglycosylated protein (D), while the 29–30 kDa and 27–28 kDa bands correspond to the M.W. of the monoglycosylated (M) and unglycosylated (U) proteins respectively. *: Non-specific signal.

The PrP protein encounters various post-translational modifications, including truncation of signalling peptides, GPI anchoring, disulfide bond formation, N-glycosylation, and α- and β- physiological cleavage. PrP^C^ is normally glycosylated at positions 182 and 198 and different paradigms show that glycans modulate the conversion of PrP^C^ into PrP^Sc^ [16], [17]. For this reason, we investigated potential differences in PrP^C^ glycosylation between the three genotypes by performing Western Blots (WB) before and after PrP^C^ deglycosylation with PNGase. Before deglycosylation, both SAF32 (aa: 78–91) and 3F4 (aa: 109–112) revealed similar PrP patterns, composed of three bands (Fig. 1): a dominant 35–37 kDa band corresponding to the diglycosylated full-length PrP^C^, and two faint bands at 29–30 kDa and 27–28 kDa, corresponding to the monoglycosylated and unglycosylated PrP^C^ isoforms respectively [17]. 8G8 antibody recognized two major bands- corresponding to diglycosylated and unglycosylated PrP^C^ - and also a faint band corresponding to the monoglycosylated form (aa: 95–110) (Fig. 1). The C-terminal antibody Pri 917 (aa: 216–221) showed a major diglycosylated band, and two faint bands corresponding to monoglycosylated and unglycosylated ptoteins (Fig. 1).The α-tubuline signal intensity of the samples agrees with the normalization realized by using 0.5X10^6^ cells per lane corresponding to 50 µg of total protein.

In addition, we observed on blot Pri917 a prominent band between the unglycosylated and monoglycosylated bands. We identified this band as non-specific, firstly because the Pri 917 (aa: 216–221) antibody is not purified but an ascitic fluid extract, and secondly because this band is not present in the 3F4 (aa: 109–112) and 8G8 blots (aa: 95–110). Finally, we recently ran a blot on PBMCs using the 12F10 (aa: 142–160), antibody which identified both C1 and C2 fragments. No non-specific band was detected (results not shown)

We calculated the ratio between diglycosylated (35–37 kDa) and unglycosylated (27–28 kDa) isoforms for each sample, after quantifying SAF32 blot bands using the Sigmagel analyzer Software. We performed ratio calculations on the SAF32 blot, because we observed better resolution of low density bands with this antibody. Comparing the averages of the three ratios, from five samples of each genotype, showed no significant difference in expression (observed t-value less than the 0.05 critical t-value) between the groups (Table 2). These findings were confirmed by quantifying the Pri 917 (C-terminus region) and 8G8 blots (middle region among 3F4). Nevertheless, the absolute ratio values differed according to the antibody used (Table 2).

### PrP^C^ cleavage studies

**Figure 2:**
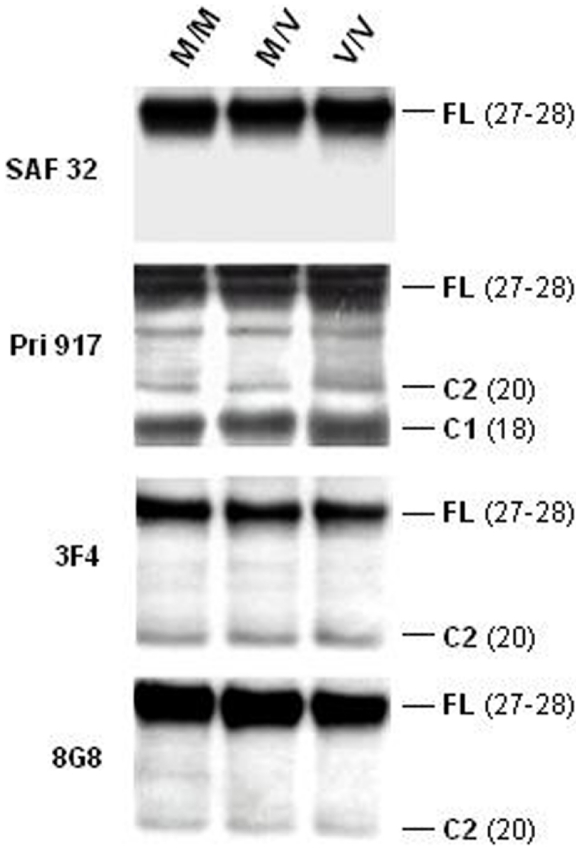
PrPC cleavage analysis. Immunoblots of truncated proteins from the three PRNP codon 129 genotypes in PBMCs. After PNGase treatment, denatured lysates from 10^6^ PBMCs were tested by Western Blot analysis using 4 antibodies to span the large PrP region: SAF32 (79–91), 8G8 (95–110), 3F4 (109–112) and PRI 917 (216–221). The 27–28 kDa band corresponds to the M.W. of the unglycosylated Full-Length (F.L.) protein, while the bands near 20 kDa and 18 kDa correspond to the C2 and C1 fragments, respectively.

In the normal brain, PrP^C^ proteolysis generates an N-terminal truncated protein - the C1 fragment - from α-cleavage at position 111/112 [18], [52]. However, β-cleavage at residue 90 can generate [52] an additional, longer C-terminal protein – the C2 fragment - that accumulates in prion-infected brain [4]. Importantly, C2 fragments correspond to protease-resistant and insoluble PrP^Sc^. We therefore investigated potential differences in PrP cleavage that might affect PrP^Sc^ generation. Following deglycosylation, we noticed identical patterns with the different anti-PrP antibodies between the three genotypes. SAF32 (aa: 78–91) recognized a major band at 27–28 kDa, which corresponds to the estimated MW of the unglycosylated full-length PrP^C^ [52]. As expected, with this N-terminal antibody, no truncated C-terminal forms could be identified (Fig. 2). Both 3F4 and 8G8 antibodies, whose epitopes are located between the α and β cleavage sites, demonstrated similar patterns: a major 27–28 kDa unglycosylated full-length protein band and a faint band near 20 kDa corresponding to the MW of the C2 NH2-terminal truncated fragment of PrP^C^ protein(Fig. 2). This confirms previous results [52] As expected, only the C-terminal antibody Pri 917 (aa: 216–221) identified both the C1 and C2 truncated proteins [52], at approximately 18 kDa and 20 kDa respectively. It also identified the major, unglycosylated full-length protein (27–28 kDa) (Fig. 2).

We calculated the ratio of the unglycosylated full-length PrP^C^ (27–28 kDa) to the C2 NH_2_-terminal truncated protein for each genotype. Even though the absolute ratios from the 8G8 and 3F4 blots were slightly different, we found no significant difference between the M/M, M/V and V/V populations (five samples per genotype) (observed *t*-value less than 0.05) for C2 expression (Table 2). As Pri 917 antibody recognizes both C1 and C2 fragments, we also compared the C1/C2 ratios. Values for the M/M, M/V and V/V populations (five samples per genotype) showed no significant difference in expression (*t*-value less than 0.05) with a good coefficient of variation (Table 2).

### Comparative study with human brain tissue (Fig. 3)

**Figure 3:**
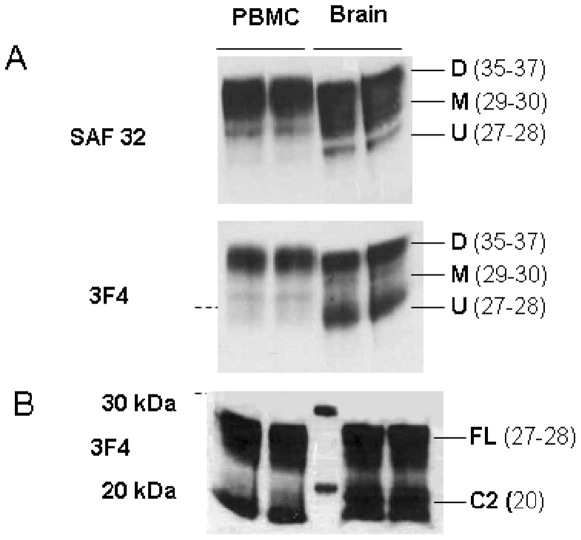
Comparative biochemical analysis between brain and PBMCs. A) Immunoblots of PrP^C^ glycoforms from PBMCs and brain, Denatured lysates from 10^6^ PBMCs and 10% brain were tested by Western Blot analysis using 2 antibodies: SAF32 (79–91), 3F4 (109–112). The 35–37 kDa band corresponds to the M.W. of the diglycosylated protein (D), while the 29–30 kDa and 27–28 kDa bands correspond to the M.W. of the monoglycosylated (M) and the unglycosylated (U) proteins, respectively. B) Immunoblots of truncated proteins from PBMCs and brain, After PNGase treatment, denatured lysates from 10^6^ PBMCs and brain were tested by Western Blot analysis using 3F4 (109–112). The 27–28 kDa bands correspond to the M.W. of the unglycosylated Full-Length (F.L.) protein while the band near 20 kDa corresponds to the C2 fragment.

We observed two differences when examining normal human brain with the same antibodies (SAF32 and 3F4). First, brain tissue showed higher levels of monoglycosylated and unglycosylated isoforms (Blots SAF 32 and 3F4, respectively), than PBMCs (fig. 3A). Secondly, brain tissue showed the C2 fragment after deglycosylation more strongly than PBMCs (fig. 3B). As the antibodies recognize the same epitope, we cannot explain variations in expression by differences in antibody affinity. These findings agree with previous studies [18], [52]. Our results suggest that both the C2 fragment and under- glycosylated PrP^C^ are under-expressed in PBMCs in comparison to brain tissue. This may result from different physiological functions or protein processing and may influence prion propagation in these tissues. We suggest that low levels of under-glycosylated isoforms and C2 fragments in PBMCs, compared to normal human brain, explains the lower blood levels of PrP^Sc^ measured when contamination does occur [53], [54].

So, the molecular mechanism explaining the relationship between the PrP M129V genotype and disease development remains elusive. Although studies have identified some specific amino-acid interactions for the methionine genotype *in vitro* [55], NMR analysis indicated no PrP^C^ instability related to M129V polymorphism that could explain disease susceptibility [56]. On the other hand, a recent study using molecular dynamic technology demonstrated a higher stability in methionine variants than valine variants [57]. In addition, a comparison of the misfolding pathway, leading to the formation of oligomeric isoforms rich in β-sheets, revealed that methionine 129 human prion protein oligomerized more rapidly than the valine 129 variant [56]. In patients with iatrogenic CJD from contaminated human growth hormone, M129V polymorphism also influences outcome. In this case, homozygous M/M and V/V patients had a shorter incubation period than the heterozygous patients [58], [59].

Taken together, our data suggest that in PBMCs, M129V PrP polymorphism has no significant effect on either PrP mRNA or protein expression. Neither processing nor apparent PrP glycoform distribution seem affected by this M129V polymorphism, which suggests that we should investigate its relationship with prion propagation elsewhere.
